# Comparison of peripheral leukocyte parameters in patients receiving conventionally and hypofractionated radiotherapy schemes for the treatment of newly diagnosed glioblastoma

**DOI:** 10.3389/fimmu.2023.1284118

**Published:** 2023-10-26

**Authors:** Lindsey Greenlund, Ryan Shanley, Kellen Mulford, Elizabeth C. Neil, Jessica Lawrence, Susan Arnold, Michael Olin, G. Elizabeth Pluhar, Andrew S. Venteicher, Clark C. Chen, Clara Ferreira, Margaret Reynolds, L. Chinsoo Cho, Christopher Wilke, B. Aika Shoo, Jianling Yuan, Kathryn Dusenbery, Lawrence R. Kleinberg, Stephanie A. Terezakis, Lindsey Sloan

**Affiliations:** ^1^ Department of Radiation Oncology, University of Minnesota, Minneapolis, MN, United States; ^2^ Clinical and Translational Science Institute, University of Minnesota, Minneapolis, MN, United States; ^3^ Masonic Cancer Center, University of Minnesota, Minneapolis, MN, United States; ^4^ Department of Neurology, University of Minnesota, Minneapolis, MN, United States; ^5^ Department of Veterinary Clinical Sciences, University of Minnesota, St. Paul, MN, United States; ^6^ Department of Pediatrics, University of Minnesota, Minneapolis, MN, United States; ^7^ Department of Neurosurgery, University of Minnesota, Minneapolis, MN, United States; ^8^ Department of Radiation Oncology and Molecular Radiation Sciences, Johns Hopkins University School of Medicine, Baltimore, MD, United States

**Keywords:** fractionation, leukocytes, hypofractionated radiotherapy, conventionally fractionated radiotherapy, glioblastoma

## Abstract

**Introduction:**

Treatment for glioblastomas, aggressive and nearly uniformly fatal brain tumors, provide limited long-term success. Immunosuppression by myeloid cells in both the tumor microenvironment and systemic circulation are believed to contribute to this treatment resistance. Standard multi-modality therapy includes conventionally fractionated radiotherapy over 6 weeks; however, hypofractionated radiotherapy over 3 weeks or less may be appropriate for older patients or populations with poor performance status. Lymphocyte concentration changes have been reported in patients with glioblastoma; however, monocytes are likely a key cell type contributing to immunosuppression in glioblastoma. Peripheral monocyte concentration changes in patients receiving commonly employed radiation fractionation schemes are unknown.

**Methods:**

To determine the effect of conventionally fractionated and hypofractionated radiotherapy on complete blood cell leukocyte parameters, retrospective longitudinal concentrations were compared prior to, during, and following standard chemoradiation treatment.

**Results:**

This study is the first to report increased monocyte concentrations and decreased lymphocyte concentrations in patients treated with conventionally fractionated radiotherapy compared to hypofractionated radiotherapy.

**Discussion:**

Understanding the impact of fractionation on peripheral blood leukocytes is important to inform selection of dose fractionation schemes for patients receiving radiotherapy.

## 1 Introduction

Glioblastoma is a devastating diagnosis with 5-year survival rates of only 5% (1). Treatment options include combinations of surgical resection, chemotherapy, and radiation therapy, conferring a 14.6-month median survival rate (2). Radiotherapy, chemotherapy, and the glioblastoma’s microenvironment negatively impact the systemic immune system (3–5). Myeloid cells, including some monocyte populations, are recruited from the peripheral blood to the tumor microenvironment to promote tumor progression (3–5). Hughes et al. were among the first to identify the prognostic relevance of radiation-related peripheral blood leukocyte disturbances specifically in patients with glioblastoma (6). In a cohort of patients receiving radiotherapy alone in the pre-Stupp therapy era, decreased lymphocyte counts led to increased susceptibility to opportunistic infection by pneumocystis carinii and was associated with shorter survival (6). A later study addressed serial changes in lymphocyte populations in patients being treated with concurrent conventionally fractionated radiotherapy and temozolomide and demonstrated persistent lymphopenia over the 9-month period studied (7). This research and others identified that radiotherapy-induced lymphopenia contributed to severe immune suppression that was detrimental to survival in patients with glioblastoma receiving chemoradiotherapy (7–9). This early work highlighted the potential role for peripheral leukocyte populations to be prognostic biomarkers in glioblastoma.

Other peripheral leukocyte populations have more recently been considered as candidate biomarkers. Since immunosuppressive macrophages dominate the glioblastoma microenvironment, it is not surprising that their myeloid cell precursors, including populations of monocytes and myeloid-derived suppressor cells, have been the focus of much research (10). Immune suppressive mechanisms of myeloid cells include depletion of L-arginine which is necessary for T cell and natural killer cell survival and function, and inhibition of anti-tumor response by T cells through cytokine signaling (11–13). In patients with glioblastoma, our group and others have identified high levels of peripheral blood leukocytes within the myeloid lineage to be associated with poor outcomes for patients on anti-neoplastic therapy (14–16). What is not understood is how significant elements of standard radiotherapy, like the dose fractionation scheme, impact peripheral blood leukocyte populations.

The standard radiotherapy approach for patients newly diagnosed with glioblastoma consists of a conventionally fractionated radiotherapy course of 6000 cGy in 30 fractions over six weeks. Hypofractionated radiotherapy paradigms are more commonly being selected due to both biological advantages and convenience for patients (17). The efficacy of hypofractionated radiotherapy has mainly been tested in elderly and/or frail patient cohorts, which make this treatment option a routine consideration as glioblastoma has a peak incidence in older aged individuals (17). Hypofractionated dose schemes recommended in the American Society for Radiation Oncology Consensus Guidelines for the treatment of select patients with glioblastoma include 4005 cGy in 15 fractions, 3400 cGy in 10 fractions, and 4500 cGy in 10 fractions (18–21). With increased dose per fraction in hypofractionated regimens, there is the potential for increased tumor cell death and subsequently higher anti-tumor efficacy, while the shorter overall course may limit peripheral lymphocyte depletion (22). Despite these advantages, tumor control following hypofractionated treatment are not superior to conventionally fractionated radiotherapy regimens, and one potential reason may be due to altered immune effects that more profoundly contribute to immunosuppression. Determining the differential peripheral immune response induced from alternative dose fractionation schedules may therefore further shape dose and fractionation regimens for future clinical practice.

One clinical laboratory test that assesses the status of the systemic immune system is complete blood count (CBC) with differential. A CBC with differential quantifies the absolute number and the percent of a leukocyte populations out of total white blood cells (WBCs) in a given peripheral blood sample. To characterize the effects of dose fractionation on systemic immunity, we analyzed the impact of conventionally fractionated and hypofractionated radiotherapy on leukocyte parameters from clinical CBC with differential tests through retrospective chart review in glioblastoma patients treated with chemoradiotherapy.

## 2 Materials and methods

### 2.1 Patients

This study was approved by the University of Minnesota Institutional Review Board (#00003179). The charts of all adult patients diagnosed with a glioblastoma and who completed radiotherapy in the Fairview Health System were identified by the Best Practices Integrated Informatics Core at the University of Minnesota. For patients who have undergone multiple rounds of radiotherapy, only the first course of radiotherapy was assessed. Individuals who did not complete the first course of radiotherapy, previously received radiotherapy for a diagnosis other than glioblastoma, were treated with brachytherapy, or who were under the age of 18 at diagnosis were excluded. Conventionally fractionated radiotherapy schemes were defined as those that delivered 180-200 cGy per fraction. Hypofractionated radiotherapy was defined as radiation courses with fraction sizes greater than 200 cGy per fraction and consisted of 15 or fewer fractions.

Data collected from patient charts included: the date of diagnosis, tumor location, pathologic characteristics, laboratory results, resection dates, radiotherapy dates and scheduling, and chemotherapy cycles. CBC data were extracted and organized by treatment time point: following diagnosis and prior to surgery, post-surgery and prior to adjuvant treatment, during adjuvant treatment, or following adjuvant treatment. Baseline CBC data for each patient was considered the time after surgery but prior to chemoradiotherapy start. Total WBCs, percent monocytes, percent lymphocytes, percent neutrophils, percent eosinophils, percent basophils, absolute monocytes, absolute lymphocytes, absolute neutrophils, absolute eosinophils, and absolute basophils were collected via CBC with differential. WBC subtypes were measured and reported using standardized units of x1000/mm^3^ cells per liter.

### 2.2 Statistical methods

**Figure 1:**
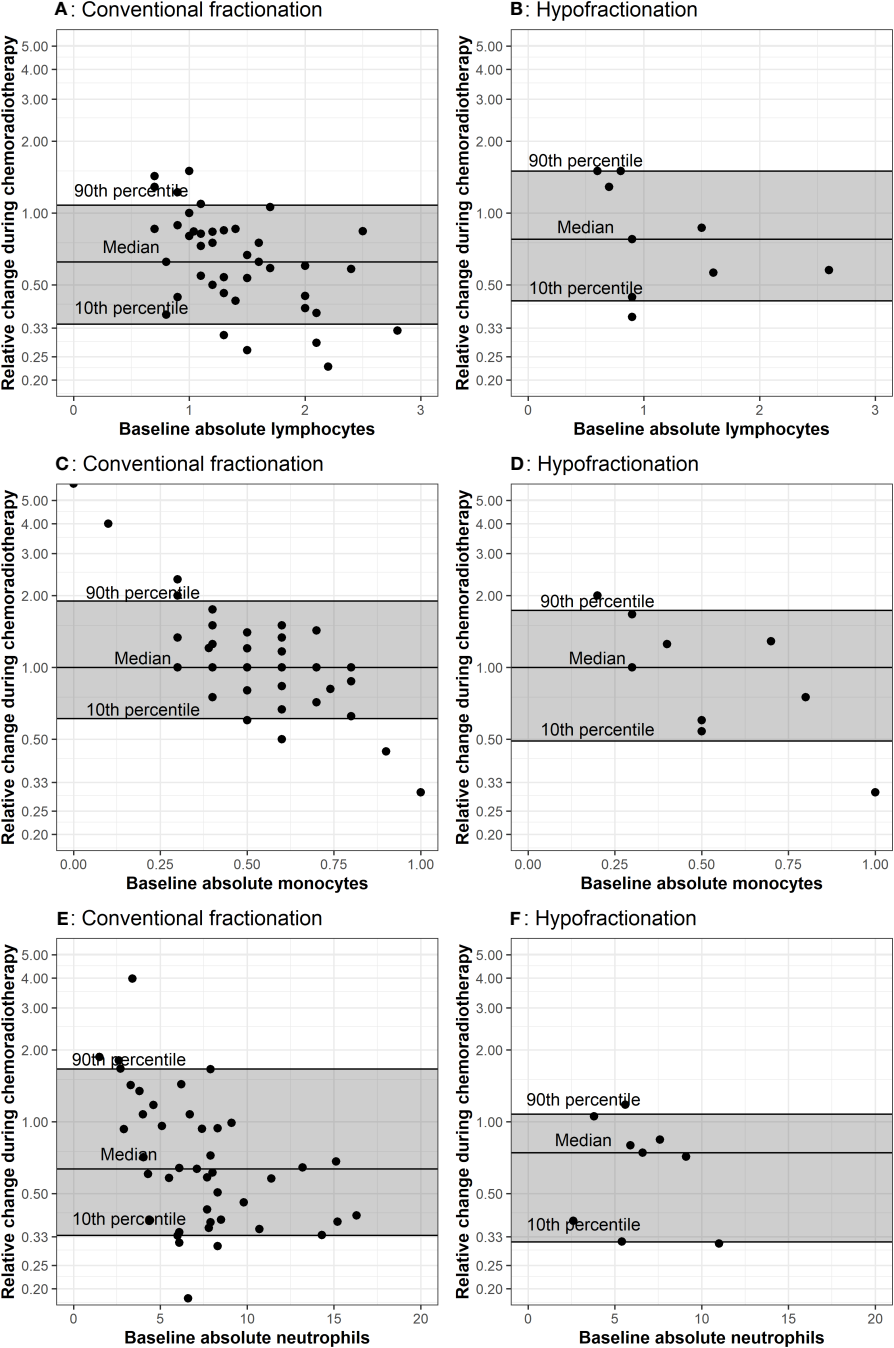
Comparison of baseline CBCs to values during chemoradiation. The baseline measurement is plotted on the x-axis because propensity for positive or negative change is affected by the baseline value, as demonstrated in A through F. The horizontal lines represent the 10^th^, 50^th^, and 90^th^ percentiles of relative change among all patients in the corresponding treatment group. Each dot represents values from one patient. Plots **(A, B)** represent lymphocyte counts in conventional and hypofractionated radiotherapy schemes. Plots **(C, D)** represent monocytes counts in conventional and hypofractionated radiotherapy schemes. Plots **(E, F)** represent neutrophil counts in conventional and hypofractionated radiotherapy schemes. WBC subtype values are presented in the form of x1000/mm^3^ cells per liter.

**Figure 2:**
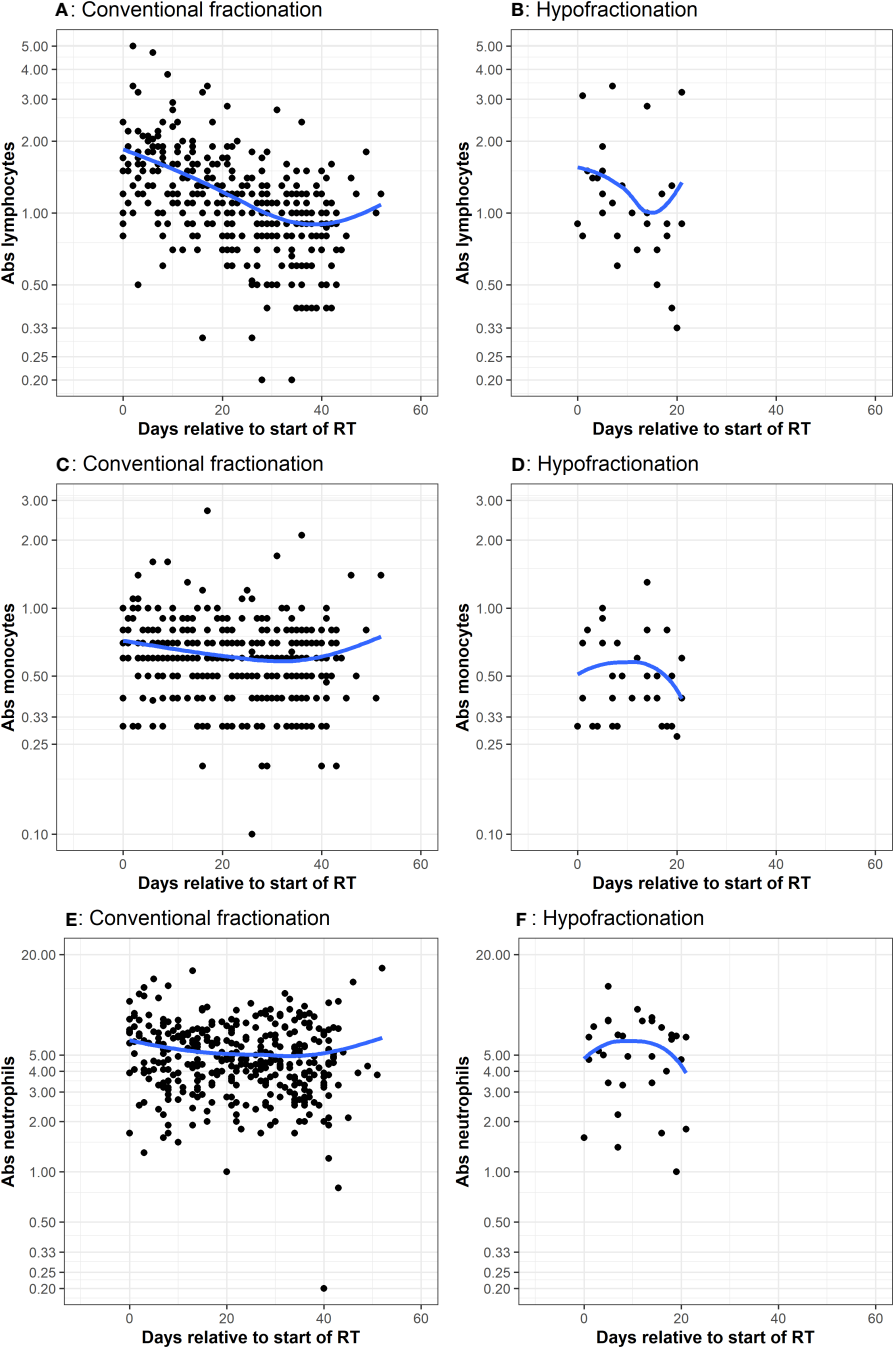
CBC measurements taken during adjuvant chemoradiation Many patients had multiple CBCs, with values corresponding to the dots. The x-axis represents days since start of RT. The y-axis is the cell count. The blue line represents a smoothed non-parametric trendline that visually describes variation of the mean of y with x. If there is no association between x and y, then trendline will be approximately a horizontal line. Plots **(A, B)** represent lymphocyte counts in conventional and hypofractionated radiotherapy schemes. Plots **(C, D)** represent monocytes counts in conventional and hypofractionated radiotherapy schemes. Plots **(E, F)** represent neutrophil counts in conventional and hypofractionated radiotherapy schemes. WBC subtype values are presented in the form of x1000/mm^3^ cells per liter.

**Figure 3:**
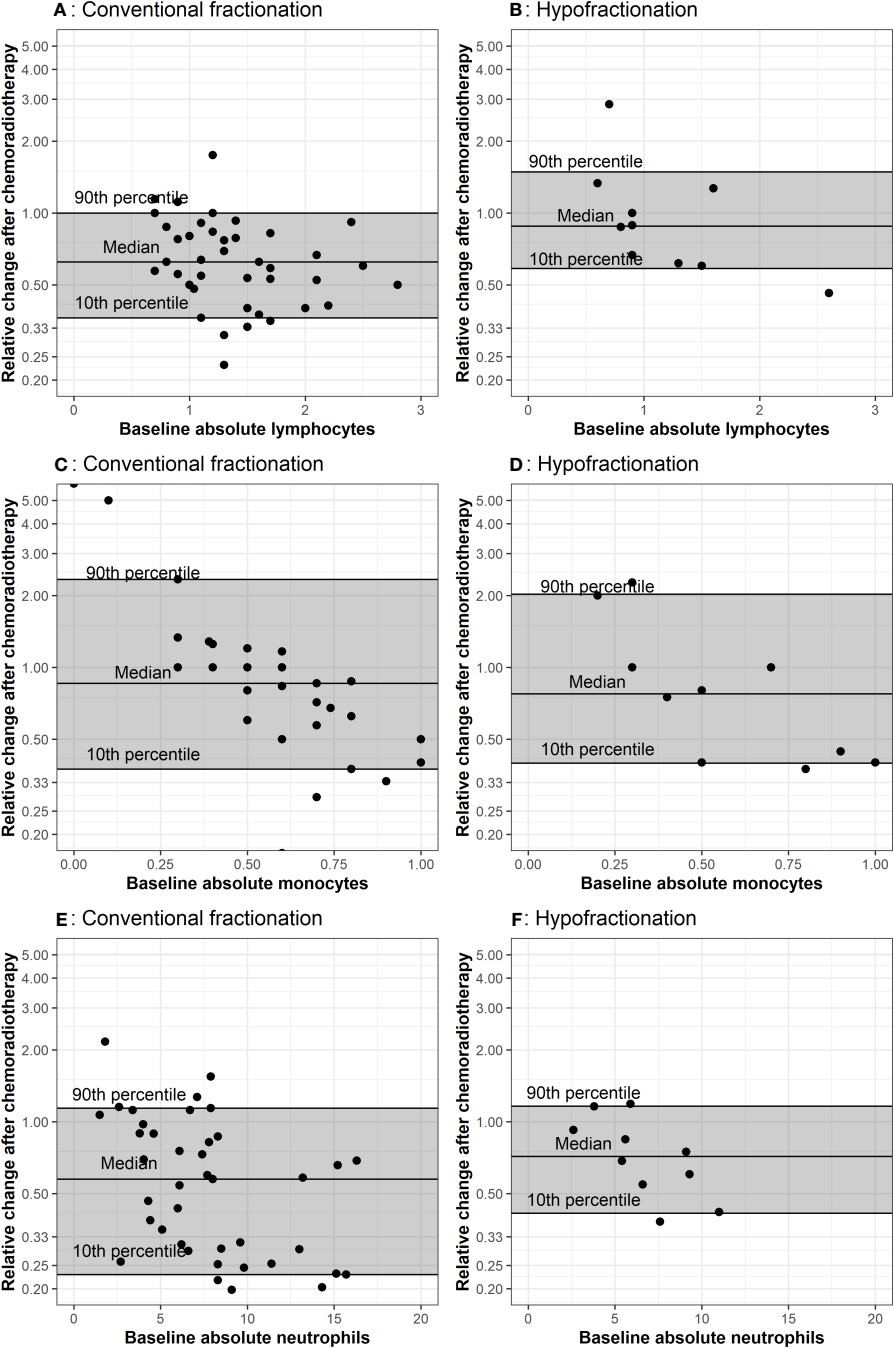
Comparison of baseline CBCs to values after chemoradiation The baseline measurement is plotted on the x-axis. The horizontal lines represent the 10^th^, 50^th^ and 90^th^ percentiles of relative change among all patients in the corresponding treatment group. Plots **(A, B)** represent lymphocyte counts in conventional and hypofractionated radiotherapy schemes. Plots **(C, D)** represent monocytes counts in conventional and hypofractionated radiotherapy schemes. Plots **(E, F)** represent neutrophil counts in conventional and hypofractionated radiotherapy schemes. WBC subtype values are presented in the form of x1000/mm^3^ cells per liter.

Within conventional and hypofractionated groups, mean pre-to-post treatment differences were calculated along with 95% paired t-confidence intervals (CI) for the mean difference. Individual changes in CBC parameters were described graphically in **Figures 1**, **3**, with relative change defined as the simple ratio of the follow-up to baseline measurement. The plots in **Figure 2** use a loess-smoothed trendline to describe the mean cell count as a function of time, using the R ggplot2 code package (23). Linear regression models were fit to compare cell counts between conventional and hypofractionated groups during and after chemoradiotherapy, including an effect for the baseline or pre-chemoradiotherapy cell count. Some cell types (total WBC, absolute monocytes, absolute lymphocytes, and absolute neutrophils) were log-transformed due to skewed distributions. Thus, the treatment effect estimates shown in **Table 2** for those cell types represent fold-differences in geometric means between the treatment groups; for remaining cell types, the effect estimate is the absolute difference in arithmetic means. We used R software version 4.0.5. for all analyses.

## 3 Results

### 3.1 Patients

Patient characteristics are described in **Table 1**. During the period from 7/1/2011 to 12/31/2020, there were 119 patients who presented for treatment of newly diagnosed glioblastoma within our health system. A total of 87 patients had available CBCs and were included in analyses. All radiotherapy was delivered with photons and all but two patients who were treated with radiotherapy received concurrent temozolomide. The median conventionally fractionated total dose received was 6000 cGy and was 4005 cGy for the hypofractionated radiotherapy group.

**Table 1:** Patient demographics.

Characteristic	Conventional Fractionation (N=72)	Hypofractionation (N=15)	Overall (N=87)
**Age in years at diagnosis, Median (Q1,Q3)**	56 (46, 63)	69 (64, 74)	59 (47, 66)
Sex			
Male (%)	43 (60%)	7 (47%)	50 (57%)
Female (%)	29 (40%)	8 (53%)	37 (43%)
Non-binary (%)	0%	0%	0%
**Radiation Intent** Definitive Adjuvant	7 (10%) 65 (90%)	4 (27%) 11 (73%)	11 (13%) 76 (87%)
**Total delivered dose in cGy, Median (min, max)**	6000 (5940, 7600)	4005 (2500, 5000)	

### 3.2 Lab value results

Baseline laboratory values were collected a median of eight days prior to the start of chemoradiotherapy for conventionally fractionated radiotherapy cases and a median of seven days prior to patients beginning hypofractionated schemes. In comparisons between baseline values to values obtained during radiotherapy, the last laboratory value collected during chemoradiotherapy was used, which was a median of treatment day 40 and 18 for conventionally fractionated and hypofractionated radiotherapy cases, respectively. In comparisons between baseline values and post-treatment values, the first laboratory value collected post-chemoradiotherapy was used, which occurred a median of 234 days and 88 days after start of treatment for conventionally fractionated radiotherapy and hypofractionated radiotherapy, respectively.

Comparing patient baseline laboratory values to values following chemoradiation, total WBCs decreased by a mean of 4.8 (95% CI -5.9, -3.7) and 1.9 (-3.8, 0.1) x1000/mm^3^ cells per liter for conventionally fractionated radiotherapy and hypofractionated radiotherapy, respectively. Absolute lymphocytes decreased by 7.0 (-8.7, -5.8) and 5.3 (-7.1, -3.5) x1000/mm^3^ cells per liter for patients receiving conventionally fractionated radiotherapy and hypofractionated radiotherapy, respectively. The percent lymphocytes increased by 1.1% (-1.8%, 4.1%) x1000/mm^3^ cells per liter for conventionally fractionated radiotherapy and by 2.1% (-3.9%, 8.0%) for hypofractionated radiotherapy. Absolute monocyte counts decreased by a mean of 0.1 (-0.2, -0.0) for conventionally fractionated radiotherapy and 0.1 (-0.3, 0.1) for hypofractionated radiotherapy. The percent monocytes increased by 2.7% (1.3%, 4.1%) in conventionally fractionated radiotherapy and increased by 0.2% (-2.2%, 2.6%) in the hypofractionated radiotherapy group. Patients who received conventionally fractionated radiotherapy had the greatest increase in the percentage of monocytes out of the WBC lineages while the percentage of eosinophils increased the most in patients receiving hypofractionated radiotherapy.

The relative change between peripheral WBC populations from baseline (i.e. post-surgery) to the last measurement during radiotherapy for each patient is depicted in **Figure 1**. Throughout radiotherapy, absolute lymphocytes decreased for the majority of patients receiving radiotherapy, regardless of fractionation. Absolute neutrophils also decreased for both groups; however, there was a greater decrease for the group receiving hypofractionated radiotherapy. Absolute monocytes remained stable for both groups throughout treatment, but a greater percentage (44%) of patients receiving hypofractionated radiotherapy experienced a decrease throughout treatment compared to patients treated with conventional fractionation (36%). There were no notable or significant changes in eosinophil or basophil populations.

Multiple CBCs for each patient throughout radiotherapy are presented in **Figure 2**. Lymphocytes decreased a similar amount for both groups. Absolute neutrophils and monocytes experienced a subtle decrease and then rebounded toward their initial values in patients receiving conventionally fractionated radiotherapy. For patients who underwent hypofractionated radiotherapy, neutrophils and monocytes experienced a brief increase and then declined. Relative change in peripheral WBC populations from baseline (i.e. post-surgery) to the first post-chemoradiotherapy measurement are depicted in **Figure 3**. Absolute neutrophils decreased for both groups with a greater decrease seen in those receiving conventionally fractionated radiotherapy. Absolute lymphocytes decreased for patients receiving conventionally fractionated radiotherapy and remained stable for patients receiving hypofractionated radiotherapy. Absolute monocytes remained relatively stable for those receiving conventionally fractionated radiotherapy and decreased slightly for the patients receiving hypofractionated radiotherapy. Percent neutrophils and percent lymphocytes were stable for both groups. Percent monocytes increased more in patients receiving conventionally fractionated radiotherapy group relative to hypofractionated radiotherapy.

Model-estimated, predicted differences in the post-radiotherapy value following hypofractionated radiotherapy relative to conventionally fractionated radiotherapy are presented in **Table 2** in the form of H:C ratios. This model predicts the difference in expected values during chemoradiotherapy (left column) or post-chemoradiotherapy (right column) if two patients with the same baseline WBC value were treated differently, specifically one received hypofractionated radiotherapy and the other received conventionally fractionated radiotherapy. Considering all peripheral blood cell types, patient monocyte counts are more likely to be affected than other cell counts by a change in fractionation scheme.

**Table 2:** Model-estimated expected differences in the follow-up measurements after hypofractionated radiation relative to conventionally fractionated radiation (H:C) adjusting for the baseline value.

	During Chemoradiotherapy	After Chemoradiotherapy
	H:C Predicted Fold Difference (95% CI)	H:C Predicted Fold Difference (95% CI)
**Total WBC**	0.99 (0.74, 1.32)	1.27 (0.97, 1.65)
**Absolute Neutrophils**	0.86 (0.57, 1.31)	1.30 (0.89, 1.92)
**Absolute Lymphocytes**	1.06 (0.79, 1.42)	1.36 (1.02, 1.81)
**Absolute Monocytes**	0.80 (0.62, 1.02)	0.88 (0.63, 1.21)
	**H:C Predicted Absolute Difference** **(95% CI)**	**H:C Predicted Absolute Difference** **(95% CI)**
**Absolute Eosinophils**	-0.04 (-0.14, 0.06)	0.07 (-0.01, 0.15)
**Absolute Basophils**	0.00 (-0.03, 0.03)	0.01 (0.00, 0.03)
**Percent Neutrophils**	-3.2 (-11, 5.1)	1.4 (-5.6, 8.4)
**Percent Lymphocytes**	3.8 (-1.2, 8.8)	-0.04 (-5.1, 5.0)
**Percent Monocytes**	-0.57 (-3.3, 2.2)	-2.8 (-5.3, -0.21)
**Percent Eosinophils**	-0.94 (-3.1, 1.2)	0.43 (-0.91, 1.8)
**Percent Basophils**	-0.01 (-0.49, 0.47)	0.13 (-0.12, 0.37)

Blue fields report predicted fold difference. Orange fields report predicted absolute difference. No change is equivalent to a 1-fold difference or 0 absolute difference.

## 4 Discussion

To the authors’ knowledge, this is the first study to compare peripheral blood leukocyte parameters between dose fractionation schemes in patients with glioblastoma. Due to known and theoretical biologic advantages of hypofractionation, we hypothesized that conventionally fractionated radiation therapy would result in higher post-chemoradiotherapy monocyte counts compared to hypofractionated radiation therapy. We found that in our cohort, hypofractionated radiotherapy and conventionally fractionated radiotherapy resulted in no change following chemoradiation in median absolute monocyte counts. Direct comparison of percent monocytes before and after chemoradiotherapy identified an increase of 2.8% in those receiving conventionally fractionated radiotherapy and a 1.6% decrease in those receiving hypofractionated treatment. The difference in absolute monocytes and percent monocytes could be due to the reliance of percent change on other leukocyte populations that are factored into the CBC with differential test. However, modeling of our data revealed a predicted fold differences in H:C ratios below 1 for absolute monocyte counts for changes from baseline to time points during chemoradiation and time points after adjuvant chemoradiation (**Table 2**). This data suggests that absolute counts of monocytes are predicted to be lower in those receiving shorter courses of radiotherapy. The H:C ratio for predicted absolute difference in percent monocyte counts for patients receiving hypofractionated radiotherapy was also predicted to be lower in patients receiving hypofractionated radiotherapy. These model findings are congruent with direct comparison of percent monocyte median values and suggest hypofractionated radiotherapy may support a more favorable peripheral blood monocyte profile.

We also hypothesized that conventionally fractionated radiotherapy would result in a lower lymphocyte count following chemoradiation compared to hypofractionated radiation therapy. Our data shows that patients receiving shorter courses of radiotherapy appeared to have a greater recovery of absolute lymphocytes relative to patients receiving conventionally fractionated radiotherapy (**Figure 3A**). These data suggest perhaps hypofractionated radiotherapy allows for a more robust recovery of the lymphocyte compartment which is critical for systemic anti-tumor immunity in glioblastoma. Modeling similarly predicted that hypofractionated schedules would result in less treatment-related lymphopenia compared to conventional fractionation (**Table 2**). This is not surprising, as it has been theorized that repeat irradiation of the circulating blood, as in cranially-directed radiotherapy for glioblastoma, may be particularly toxic to the lymphocyte compartment (24).

Determining the etiology of these identified leukocyte parameters will be an import next step. As cranial radiotherapy promotes mobilization of immature myeloid cells from bone marrow in animal models of glioblastoma, one possible explanation is that fractionation affects the rate of tissue trafficking (25). The fate WBC subsets could be further studied with immunocompetent pre-clinical animal models of glioblastoma. Interestingly, the H:C difference of percent neutrophils during chemoradiotherapy was -3.2, predicting less of this cell type in hypofractionated patients during chemoradiotherapy compared to those receiving conventionally fractionated courses, but positive (1.4) after chemoradiotherapy, suggesting the inverse relationship (**Table 2**). The temporal difference between values during chemoradiotherapy compared to values after chemoradiotherapy in absolute neutrophil H:C ratio is unclear and may reflect the role of neutrophils in acute inflammation. The “call” of neutrophils to circulate in the blood in patients with glioblastoma who are receiving radiotherapy may be greater with larger fraction sizes, as seen in hypofractionated radiotherapy. Like myeloid cells, a large number of circulating tumor-associated neutrophils is a poor prognostic indicator, suggesting maybe the resulting inflammation from a hypofractionated course of radiotherapy delivered to the brain should not be sustained if optimal immunity is desired (26). Both neutrophils and monocytes have been reported to demonstrate radiotherapy resistance, potentially contributing to tumor immune system evasion (27). Complicating the use of neutrophils as biomarkers for glioblastoma is the high concomitant rate of glucocorticoid (and chemotherapy) use of patients receiving radiotherapy for brain tumors (28). Additional studies are needed to better describe and use of neutrophil kinetics in glioblastoma.

While this is the first report to describe key differences in WBC parameters between patients with glioblastoma treated with two distinct fractionation schemes, there are limitations to this retrospective study. Some caution with interpretation is warranted given the small sample size of 15 within our hypofractionated radiotherapy group and the wide range of total dose used, which may have led to type II error. Because this fractionation scheme is only recommended for select patients and many of the clinical trials that support hypofractionated protocols were published after our data collection start date, it is not surprising that the group size is notably smaller for the hypofractionated radiotherapy course (21). Despite this small sample size, baseline total WBC counts during radiotherapy, regardless of dose fractionation group, and all analyzed cell types determined by clinical CBC trended down (**Figures 1A–E**). These same findings held when considering changes from baseline to after adjuvant therapy, regardless of dose fraction scheme, as all cell lines appeared to decrease following adjuvant chemoradiotherapy (**Figures 3A–E**). These data are consistent with the robust amount of literature that also found decreases in total leukocytes in response to radiotherapy; supporting that our results may hold in larger cohorts (29, 30). Additionally, since hypofractionated radiotherapy courses are by definition shorter than conventionally fractionated courses, the duration of concurrent chemotherapy also differs between groups. Although the optimal duration of chemotherapy in glioblastoma patients continues to be under investigation, differing lengths of chemotherapy could explain the differences seen in our results (31). Lymphopenia has been identified to be a poor prognostic indicator, independent of chemotherapy in prior cohorts, which highlights the importance of data reported here. Other limitations relate to the retrospective nature of our study. Hypofractionated radiotherapy approaches are recommended to be used for patients who are elderly and aging itself is associated with altered blood counts (32). A strength of our study was the inclusion of only naïve glioblastoma patients; future prospective studies should also match for age, performance status, and co-morbidities to account for differences between cohorts. It is possible that these are confounding factors that we cannot adequately adjust for given the small sample size of the hypofractionated group in this study. Finally, our patients received the majority of their treatments at a single system; a multi-center approach provide more data and ensure our findings are reflective of the populations of patients with glioblastoma outside of our institution.

We identified trends in leukocyte parameters that support further exploration into the distinct biologic effects of differing radiotherapy dose fractionation schemes. Additional translational studies assessing the phenotype and activation status of peripheral leukocytes in patients with glioblastoma who are receiving radiotherapy are actively being investigated by our group and others (33, 34). Future projects should include greater numbers of patients and/or utilize animal modeling to determine the fate of leukocyte cell lineages and distinguish effects of radiotherapy from chemotherapy. A better understanding of the effect of radiation dose fractionation on leukocyte parameters may lead to the informed selection of dose fractionation for future cancer patients.

In summary, we assessed the impact of hypofractionated radiotherapy compared to conventionally fractionated radiotherapy for newly diagnosed glioblastoma on systemic immunity via clinically available peripheral blood counts. In our patient cohort, hypofractionated radiotherapy and conventionally fractionated radiotherapy resulted in similar peripheral leukocyte profiles. However, notable but non-significant trends were found in both lymphocyte and monocyte cell counts suggesting that these cell linages may be altered by radiotherapy. As hypofractionated dose schedules gain favor for their convenience for patients and potential to support favorable anti-tumor immunity, it is important to fully understand their immunologic effects. Further insight into the impact of radiotherapy on leukocyte parameters in cancer patients may be crucial in the development of future therapies and in the temporal combination of systemic agents with radiotherapy.

## Data availability statement

The raw data supporting the conclusions of this article will be made available by the authors, without undue reservation.

## Ethics statement

The studies involving humans were approved by Dr. Shashank Priya, Vice President for Research, Office of the Vice President for Research. The studies were conducted in accordance with the local legislation and institutional requirements. Written informed consent for participation was not required from the participants or the participants’ legal guardians/next of kin in accordance with the national legislation and institutional requirements.

## Author contributions

LG: Data curation, Formal Analysis, Funding acquisition, Investigation, Project administration, Software, Visualization, Writing – original draft, Writing – review & editing. RS: Conceptualization, Data curation, Formal Analysis, Investigation, Methodology, Software, Supervision, Validation, Visualization, Writing – original draft, Writing – review & editing. KM: Data curation, Methodology, Software, Writing – review & editing. EN: Writing – review & editing, Conceptualization, Resources, Validation. JL: Conceptualization, Investigation, Validation, Writing – original draft, Writing – review & editing. SA: Writing – review & editing. MO: Writing – review & editing. GP: Writing – review & editing. AV: Writing – review & editing. CC: Writing – review & editing. CF: Writing – review & editing. MR: Writing – review & editing. LC: Writing – review & editing. CW: Writing – review & editing. BS: Writing – review & editing. JY: Writing – review & editing. KD: Writing – review & editing. LK: Writing – review & editing, Validation. ST: Writing – review & editing, Resources, Validation. LS: Resources, Validation, Writing – review & editing, Conceptualization, Data curation, Formal Analysis, Funding acquisition, Investigation, Methodology, Project administration, Software, Supervision, Visualization, Writing – original draft.
